# Sickle Cell Transplantation Evaluation of Long-term and Late Effects Registry (STELLAR) to Compare Long-term Outcomes After Hematopoietic Cell Transplantation to Those in Siblings Without Sickle Cell Disease and in Nontransplanted Individuals With Sickle Cell Disease: Design and Feasibility Study

**DOI:** 10.2196/36780

**Published:** 2022-07-06

**Authors:** Lakshmanan Krishnamurti, Staci D Arnold, Ann Haight, Allistair Abraham, Gregory MT Guilcher, Tami John, Nitya Bakshi, Shalini Shenoy, Karen Syrjala, Paul L Martin, Sonali Chaudhury, Gretchen Eames, Olusola Festus Olowoselu, Matthew Hsieh, Josu De La Fuente, Kimberly A Kasow, Elizabeth Stenger, Anne Mertens, Fuad El-Rassi, Peter Lane, Bronwen E Shaw, Lillian Meacham, David Archer

**Affiliations:** 1 Aflac Cancer and Blood Disorders Center Children's Healthcare of Atlanta Emory University School of Medicine Atlanta, GA United States; 2 Pediatric Hematology/Oncology/Blood and Marrow Transplantation Children's National Health System Washington, DC United States; 3 Section of Pediatric Oncology and Blood and Marrow Transplant Alberta Children's Hospital University of Calgary Calgary, AB Canada; 4 Bone Marrow Transplant / Stem Cell Transplant Program Cancer and Hematology Center Texas Children's Hospital, Baylor College of Medicine Houston, TX United States; 5 Division of Pediatric Hematology/Oncology/Bone Marrow Transplantation Washington University in St. Louis St. Louis, MO United States; 6 Clinical Research Division Fred Hutchinson Cancer Research Center Seattle, WA United States; 7 Pediatric Transplant and Cellular Therapy Duke University School of Medicine Durham, NC United States; 8 Division of Pediatric Hematology Oncology/Bone Marrow Transplantation Ann and Robert H Lurie Children's Hospital of Chicago Chicago, IL United States; 9 Division of Pediatric Hematology/Oncology/BMT Cook Children's Medical Center Fortworth, TX United States; 10 Department of Medicine and Hematology Lagos University Teaching Hospital Lagos Nigeria; 11 National Institutes of Health Clinical Center National Heart, Lung, and Blood Institute Bethesda, MD United States; 12 Division of Pediatric Hematology/Oncology/BMT Imperial College London Faculty of Medicine St. Mary's Hospital London United Kingdom; 13 Division of Pediatric Hematology Oncology University of North Carolina Chapel Hill, NC United States; 14 Department of Hematology Emory University School of Medicine Atlanta, GA United States; 15 Center for International Blood and Marrow Transplant Research (CIBMTR) Department of Medicine Medical College of Wisconsin Milwaukee, WI United States

**Keywords:** anemia, sickle cell, late effect, hematopoietic cell transplantation, web-based registry, sickle cell disease, transplant, protocol

## Abstract

**Background:**

There are sparse data on the long-term and late effects of hematopoietic cell transplantation (HCT) for sickle cell disease (SCD).

**Objective:**

This study aims to establish an international registry of long-term outcomes post-HCT for SCD and demonstrate the feasibility of recruitment at a single site in the United States.

**Methods:**

The Sickle Cell Transplantation Evaluation of Long-Term and Late Effects Registry (STELLAR) was designed to enroll patients with SCD ≥1 year post-HCT, their siblings without SCD, and nontransplanted controls with SCD to collect web-based participant self-reports of health status and practices by using the Bone Marrow Transplant Survivor Study (BMTSS) surveys, health-related quality of life (HRQOL) using the Patient-Reported Outcomes Measurement Information System (PROMIS) Pediatric Profile-25 or Pediatric Profile-29 survey, chronic graft-versus-host disease (cGVHD) using the symptom scale survey, daily pain using an electronic pain diary, the economic impact of HCT using the financial hardship survey, sexual function using the PROMIS Sexual Function SexFSv2.0 survey, and economic productivity using the American Time Use Survey (ATUS). We also piloted retrieval of clinical data previously submitted to the Center for International Blood and Marrow Transplant Research (CIBMTR); recorded demographics, height, weight, blood pressure, waist and hip circumferences, timed up and go (TUG) test, and handgrip test; and obtained blood for metabolic screening, gonadal function, fertility potential, and biorepository of plasma, serum, RNA, and DNA.

**Results:**

Of 100 eligible post-HCT patients, we enrolled 72 (72%) participants aged 9-38 (median 17) years. We also enrolled 19 siblings aged 5-32 (median 10) years and 28 nontransplanted controls with SCD aged 4-46 (median 22) years. Of the total 119 participants, 73 (61%) completed 85 sets of surveys and 41 (35%) contributed samples to the biorepository. We completed ATUS interviews of 28 (24%) participants. We successfully piloted retrieval of data submitted to the CIBMTR and expanded recruitment to multiple sites in the United States, Canada, the United Kingdom, and Nigeria.

**Conclusions:**

It is feasible to recruit subjects and conduct study procedures for STELLAR in order to determine the long-term and late effects of HCT for SCD.

**International Registered Report Identifier (IRRID):**

DERR1-10.2196/36780

## Introduction

Hematopoietic cell transplantation (HCT) remains the only treatment undertaken with curative intent for sickle cell disease (SCD). HCT has the possibility of alleviating disease-related morbidity, improving physical function, stabilizing organ function, and improving the quality of life [1,2]. The results of HCT for SCD from human leukocyte antigen (HLA)-identical sibling donors are excellent, with over 94% overall survival and 91% event-free survival [3-8]. Alternative donor HCT [5,9-13] and autologous gene therapy (GT) can further increase the applicability of HCT for SCD [9,14-16]. Observational case series, clinical trials, and research registries have typically captured the short- and intermediate-term outcomes of engraftment, graft-versus-host disease (GVHD), and survival 1-3 years post-HCT. However, the late effects of HCT, including detailed patient-reported outcomes (PROs), physical function, health status, health behaviors, and health outcomes, have not been captured. In addition, approximately 10% of post-HCT mortality after HCT for SCD occurs beyond 5 years after HCT [6]. Therefore, there is potential for persistent or new late morbidity following HCT for SCD. These observations provide a rationale for the systematic study of long-term and late effects to understand the impact of allogeneic HCT and autologous GT on patients' life course and outcomes.

The impact of HCT in SCD can be better understood by comparing the long-term outcomes in the post-HCT group with those in their siblings without SCD and in nontransplanted persons with SCD matched for age, genotype, and disease characteristics that define the propensity to undergo HCT. Siblings share social, psychological, and environmental exposures and may share genetic predispositions. Thus, this comparison group provides the best approximation of how the participants may have fared had they not been born with SCD. In contrast, nontransplanted individuals with SCD matched for age, gender, and propensity to undergo HCT provide the best estimate of what may have been the outcomes of post-HCT patients had they not undergone HCT. Unfortunately, no such contemporaneous comparison cohort has been established to date, despite the awareness of this knowledge gap.

We designed the Sickle Cell Transplantation Evaluation of Long-Term and Late Effects Registry (STELLAR) to address this knowledge gap and track and compare the long-term outcomes and late effects of HCT for SCD compared to unaffected sibling controls and nontransplanted patients with SCD. We implemented the registry in collaboration with the Center for International Blood and Marrow Transplant Research (CIBMTR) and core centers experienced in the conduct of HCT for SCD. Our overarching hypothesis is that *HCT for SCD improves the health-related quality of life (HRQOL) and immune function but is associated with gonadal damage and impaired fertility potential compared to nontransplanted controls with SCD and sibling controls without SCD*. Our objectives were to (1) compare the long-term HRQOL, pain, financial hardship, physical function, health status, health behaviors, and economic productivity; (2) compare gonadal function and fertility status in adults post-HCT for SCD with these contemporaneous comparison groups; and (3) leverage current data collected through the CIBMTR to harmonize data collection and avoid duplication of effort in the field. This report describes STELLAR’s design and development and its pilot testing and implementation in participating pediatric and adult programs in Atlanta, GA, USA.

## Methods

### Study Design

STELLAR is a prospective, longitudinal, observational tool comparing health outcomes in study participants post-HCT for SCD, siblings without SCD, and a contemporary group of nontransplanted subjects with SCD.

### Ethics

The study was approved by the Western Institutional Review Board (IRB), which served as the single IRB for the study (approval number WIRB 20200372). All the study procedures followed were in accordance with the ethical standards of the responsible committee on human experimentation (institutional and national) and with the Declaration of Helsinki of 1975, as revised in 2000.

### Participants

The inclusion criteria were (1) patients with SCD of any age >1 year post-HCT or autologous GT; (2) HLA-matched donor siblings for post-HCT participants or a sibling without SCD of the recipient who is closest in age for the recipient of the transplant from an HLA-matched unrelated donor, HLA-haploidentical related donor, or autologous GT; and (3) patients with SCD (Hemoglobin SS [HbSS] or Hemoglobin S/β0 Thalassemia [HbSβ^0^]) who have not undergone HCT.

The exclusion criterion was non-English-speaking individuals.

### Setting

The study was designed as a collaboration among several sites with substantial experience and expertise in performing HCT for SCD (Textbox 1) for the recruitment of subjects through direct contact and in clinic settings.

**Participating sites.**

- Emory University, Aflac Cancer and Blood Disorders Center, Children’s Healthcare of Atlanta, GA, USA
- Emory University Department of Hematology, Grady Hospital, Atlanta, GA, USA
- Children's National Health Systems, Washington, DC, USA
- Columbia University Medical Center, NY, USA
- Cook Children's Medical Center, TX, USA
- Alberta Children’s Hospital, Calgary, Canada
- Children's Hospital of Los Angeles, CA, USA
- Imperial College Healthcare, London
- National Heart, Lung, and Blood Institute, Bethesda, MD, USA
- University of North Carolina, NC, USA
- Lagos University Teaching Hospital, Lagos, Nigeria
- Duke University, NC, USA
- Washington University School of Medicine, MO, USA
- Baylor College of Medicine, TX, USA

### Recruitment of Individuals Post-HCT for SCD

We reviewed electronic medical records to identify individuals who underwent allogeneic HCT or autologous GT or follow-up care in the participating centers. Then, using the last-known contact information, we approached potential participants by mail, email, and telephone. For participants currently <18 years old, we approached their parents/legal guardians for participation. In the case of potential study participants ≥18 years old, we contacted them directly. When we did not have the correct contact information for potential participants, we used social media and people-finding software to establish contact with those individuals. In addition, we organized annual reunions of survivors and their families to raise awareness of the study. Participants aged ≥18 years were also offered participation in the reproductive health substudy to assess sexual function and reproductive health. Individuals post-HCT for SCD were also approached when they attended an ex-sickle clinic, a clinic designated for long-term follow-ups of patients with successful HCT for SCD.

### Recruitment of Nontransplanted Controls With SCD

After establishing procedures for enrollment of transplant recipients, we sequentially expanded enrollment to siblings without SCD and nontransplanted participants with SCD. Nontransplanted patients with SCD were approached for study in a comprehensive sickle cell clinic. Patients and siblings were also approached in annual post-HCT reunions and through social media. In the case of minor siblings, we contacted their parents to obtain informed consent. In the case of adult siblings, we received permission from HCT survivors or their parents to contact the siblings.

We piloted the enrollment of pediatric and adult nontransplanted patients with SCD in the pediatric and adult sickle cell programs in Atlanta, GA, USA. Since registration is ongoing and participants are of a wide age range, we enrolled control subjects regardless of age to establish a pool of control patients. For matching post-HCT participants to nontransplanted individuals, we will select age, gender, and the propensity score matched to post-HCT patients. We will use logistic regression for propensity score calculation from the following variables: (1) the number of episodes of acute chest syndrome, (2) the frequency of hospitalization for a vaso-occlusive crisis in the 2 years pre-HCT, and (3) a history of stroke. A 1:1 propensity score matching will be performed using the nearest-neighbor-matching method with a caliper width fixed at 0.2. Propensity score matching will be performed using JMP Pro 13.2.0 (SAS Institute Japan, Co, Ltd, Tokyo, Japan).

### Study Procedures

The study procedures included medical record review, data retrieval from the CIBMTR, participant-completed surveys and electronic pain diaries, fertility evaluation, clinical parameters, vital measurements, physical function tests, and blood samples for metabolic screening, gonadal and fertility potential, and biobanking specimens.

### Clinical Parameters and Vital Measurements

We recorded clinical parameters, including vital signs, and measurements, including height, weight, and waist and hip circumferences. In addition, we performed a handgrip test, a measure of the maximum isometric strength of hand and forearm muscles and the widely used general muscle strength [17-20], a timed up and go (TUG) test [21,22], and a test of mobility and balance as assessments of physical function [17-19]. We will repeat these procedures annually.

### Surveys

We used a set of validated surveys to capture patient reports of health outcomes, health practices, and the HRQOL (Table 1). The Bone Marrow Transplant Survivor Study (BMTSS) survey [23,24] is a measure that has been extensively validated for use in long-term survivors of BMT to capture patient reports of health, health practices, health interventions, and complications [23-25].

The patient report on this survey was validated against medical records and was found to be accurate. The BMTSS surveys contain 130 items that ask questions on health status regarding hearing, vision, speech, and urinary tract; hormonal, heart and circulatory, respiratory, digestive, and brain and nervous systems; cancer; offspring; and pregnancy. The surveys also address health habits and practices related to alcohol or substance abuse, school history, employment history, and insurance. We reduced the burden of completing BMTSS surveys by using branching logic, also known as skip logic, which creates a custom pathway based on a user's response and accordingly presents subsequent questions, thus allowing the user to skip a question that does not apply to them.

Patients <18 years old completed the Patient-Reported Outcomes Measurement Information System (PROMIS) Pediatric Profile-25 survey. This survey assesses 6 HRQOL domains (ie, mobility, anxiety, depression, fatigue, peer relationships, and pain interference) by asking 4 questions per domain [26-30]. There is a single item on pain intensity. Patients >18 years old completed the PROMIS Pediatric Profile-29 v2.0 survey [31,32], which assesses pain intensity by a single question on a 0-10 rating scale and 6 health domains (ie, physical function, fatigue, pain interference, depressive symptoms, anxiety, and ability to participate in social roles and activities) and sleep disturbance using 4 questions per domain. Adults also completed the stiffness subscale of the Adult Sickle Cell Quality of Life Measurement Information System (ASCQ-Me) [29,33-35].

All patients with SCD completed the sickle cell self-efficacy survey [26-30,36] containing 9 questions relating to participants' perceptions of their ability to function daily and manage SCD symptomatology [36,37]. In addition, transplant recipients completed the chronic graft-versus-host disease (cGVHD) symptom survey and financial hardship assessments. The cGVHD symptom scale is a 30-item scale with 7 subscales to capture the cGVHD-specific burden [38,39].

To determine the impact of HCT on financial hardship, including income, employment, and insurance status, we adapted a 43-item measure developed at the Dana Farber Cancer Institute (DFCI) and used in stem cell transplant economic impact studies [40,41]. We modified the survey to a 38-question patient-reported financial hardship assessment tool. In addition, we adapted the survey for use in a pediatric population with parent proxy and age-appropriate patient surveys.

Study participants completed surveys electronically. The application is adaptable for use on smartphones, tablets, or computers and is platform “agnostic.” Participants can start, stop, and save completion of surveys at will. To further minimize the burden of survey completion, we split the surveys into 4 parts and gave participants the option of completing these surveys quarterly over the year. To reduce the burden for completion in subsequent years, the response fields are automatically populated with draft responses from previous years. Participants are prompted to accept or change the response to proceed to the next screen. We incorporated a page timer in the surveys to track the amount of time spent by participants in each survey.

Our hypothesis was that those with SCD have lower rates of participation in economic activity and spend more time in health-related activities than the African American population in general as well as patients with SCD who are long-term survivors of HCT for SCD. To test this hypothesis, we piloted the American Time Use Survey (ATUS), a structured computer-assisted telephone interview.

**Table 1:** Surveys completed and average time taken to complete them.

Description of scale		Average time to complete
**Health and health practice surveys for all age groups (total time for the first quarter: 11 min 31 s)**		
	Demographics	1 min 17 s
	School history	1 min 7 s
	Employment history	1 min 2 s
	Insurance	51 s
	PROMIS^a^ Adult Profile v2.0 or PROMIS Pediatric Profile-25 v2.0	6 min 36 s
	ASCQ-Me^b^ stiffness	26 s
**Health and health practice surveys for all age groups (total time for the second quarter: 8 min 13 s)**		
	cGVHD^c^	1 min 38 s
	Previous encounters	45 s
	Family history	4 min 46 s
	Marital status	52 s
	Religion	7 s
**Health and health practice surveys for all age groups (total time for the third quarter: 5 min)**		
	Hearing and speech	41 s
	Urinary system	13 s
	Hormonal system	35 s
	Health and circulatory system	40 s
	Respiratory system	29 s
	Digestive system	31 s
	Brain and nervous system	1 min 53 s
**Health and health practice surveys for all age groups (>total time for the fourth quarter: 11 min 6 s)**		
	Financial survey	5 min 26 s
	Health habits	1 min 32 s
	Surgical procedures	59 s
	Medical care	1 min 19 s
	Other issues (SCD^d^ SEQ-C^e^)	22 s
	Financial survey	2 min
**Reproductive Health Survey (HCT^f^ recipients and controls with SCD aged >18 years only)**		
	PROMIS Sexual Function SEXFSv2.0	7 min
**Service utilization and cost (transplant recipients and controls with SCD)**		
	GAIN^g^ Scale	3 min
**Financial burden of HCT**		
	DFCI^h^ finances and employment scale	3 min
**ATUS^i^**		
	Economic productivity	30-45 min interview

^a^PROMIS: Patient-Reported Outcomes Measurement Information System.

^b^ASCQ-Me: Adult Sickle Cell Quality of Life Measurement Information System.

^c^cGVHD: chronic graft-versus-host disease.

^d^SCD: sickle cell disease.

^e^SCD SEQ-C: Sickle Cell Disease Self-Efficacy Questionnaire for Children.

^f^HCT: hematopoietic cell transplantation.

^g^GAIN: Global Assessment of Individual Needs.

^h^DFCI: Dana Farber Cancer Institute.

^i^ATUS: American Time Use Survey.

### Assessment of Reproductive Potential and Hormonal and Sexual Function

For post-HCT patients and nontransplanted controls with SCD who were ≥18 years old, we administered offspring and pregnancy history surveys, reproductive health from the BMTSS, and PROMIS sexual function and satisfaction surveys v2.0 [7,8]. Blood samples were collected for assay of reproductive hormones, including anti-Mullerian hormone (AMH), luteinizing hormone (LH), follicle-stimulating hormone (FSH), and estradiol in females and LH, FSH, and testosterone in males. In addition, fertility potential was assessed by semen analysis in males and the antral follicle count in females. A STELLAR study fertility specialist interpreted reproductive hormone labs, semen analysis, and antral follicle counts.

### Data Retrieval From the CIBMTR

We collaborated closely with the CIBMTR to develop a process for retrieving clinical data submitted by centers to the CIBMTR before and after HCT. The CIBMTR has now established mechanisms by which centers can recover their own submitted data, either on an individual patient level or with data visualizations. The CIBMTR has also collaborated with the CureSC initiative to prepare a deidentified publicly available data set of patients with SCD undergoing HCT. This data set includes variables relevant to late effects, approved by a large group of stakeholders, and standard pre-, peri-, and post-HCT patient, disease, and demographic variables. The CIBMTR leveraged the CureSC data set to identify patients transplanted at the Atlanta site, and data have already been successfully retrieved. The CIBMTR will facilitate a process to offer participating STELLAR locations their center code to identify their patients within the publicly available data set and merge those data with other data at their center.

### Medical Record Data Abstraction

The clinical data unavailable or not collected by the CIBMTR and relevant to this study were abstracted from the patients’ medical charts. We reviewed the medical records and collected data from the clinical assessment to determine health care utilization, disease complications, and outcomes in post-HCT patients, non-HCT patients with SCD, donors, and healthy sibling controls.

### Pain Diary

We used a validated web-based electronic multidimensional pain diary for collecting ecological momentary assessment (EMA) pain data [42]. Post-HCT participants with SCD and nontransplanted controls with SCD completed an electronic pain diary, as described earlier, twice a day for 2 weeks each year. Participants were asked to use the pain diary if they were ≥8 years old, had undergone HCT or autologous GT, or had SCD. Participants were asked to begin survey completion at their convenience and as soon as possible after study enrollment. The items on the pain diary include pain intensity, pain location, pain quality description, interference with sleep, mood, work/school, daily life, interactions with friends and family, and medications and nonpharmacological treatments for pain. There are 5 items for morning data collection and 14 items for evening data collection, which take approximately 5 min to complete.

### Biological Specimen Collection

Blood samples of subjects who consented were collected by phlebotomy during the visit. The timing of blood and urine sample collection and other study procedures is described in Table 2. Metabolic screening was implemented with fasting blood sugar levels, urinalysis, the complete blood count, and the lipid profile.

**Table 2:** Specimen collection: blood work and procedures.

Labs and procedures	Enrollment	Annual
Biorepository^a^	✓	N/A^b^
C-reactive protein	✓	N/A
Fibrinogen	✓	N/A
Troponin-I	✓	N/A
Brain natriuretic peptide (BNP)	✓	N/A
Immunoglobulin G (IgG)	✓	N/A
Fasting blood glucose	✓	✓
Glucose fructosamine	✓	✓
Insulin level	✓	✓
Urinalysis	✓	✓
Urine for microalbuminuria	✓	✓
Complete blood count with differential	✓	✓
Urine creatinine	✓	✓
Lipoprotein, serum lipids after 12 h fast	✓	✓
Immunophenotype of T, B, and natural killer (NK) cells	✓	N/A
Pneumococcal-23 serotype IgG	✓	N/A
D-dimer	✓	N/A
FSH^c^ (≥11 years old)	✓	✓
LH^d^ (≥11 years old)	✓	✓
AMH^e^ (females ≥11 years old)	✓	✓
Testosterone (males ≥11 years old)	✓	✓
Estradiol (females ≥11 years old)	✓	✓
Blood urea nitrogen (BUN)	✓	✓
Creatinine	✓	✓
Thyroid panel	✓	✓
Lactate dehydrogenase (LDH)	✓	N/A
Hemoglobin electrophoresis	✓	✓
Chimerism study (HCT^f^ patients only; fluorescent in situ hybridization [FISH] or variable number of tandem repeats [VNTR]; not paid for by study funds)	✓	✓
Semen analysis (males enrolled in reproductive health aim)	✓	N/A
Antral follicle count by vaginal ultrasound (females enrolled in the reproductive health aim	✓	N/A
Height/weight^g^	✓	✓
Hip/waist circumference^g^	✓	✓
Handgrip^h^	✓	N/A
TUG^h,i^	✓	N/A
Pain diary^j^	✓	N/A
Surveys^k^	✓	✓

^a^Biorepository specimen tests include testing of soluble urokinase plasminogen activator receptor (suPAR) and metabolomics to identify untargeted and global small-molecule metabolites, functional opsonophagocytic activity, and splenic function assay with flow cytometric enumeration of Howell-Jolly micronuclei. The biorepository specimens will be shipped to the Children’s Healthcare of Atlanta lab.

^b^N/A: not applicable.

^c^FSH: follicle-stimulating hormone.

^d^LH: luteinizing hormone.

^e^AMH: anti-Mullerian hormone.

^f^HCT: hematopoietic cell transplantation.

^g^All participants will complete height/weight and hip/waist circumference measurements.

^h^TUG: timed up and go.

^i^Participants ≥4 years old will complete handgrip and TUG testing.

^j^The pain diary will only be used over a 2-week period for individuals ≥8 years old who underwent HCT or autologous GT^l^ or have SCD^m^. Please see the Pain Diary section for additional details.

^k^Refer to Textbox 1 and Table 1 surveys for additional information.

^l^GT: gene therapy.

^m^SCD: sickle cell disease.

### Participant Tracking/Monitoring

Participants receive automated reminders for study procedures. In addition, research coordinators monitor the status of completing surveys, reach out directly to participants, and offer reminders and technical support, as needed. We will also continue the engagement of study participants through relevant educational messages on the study website, personal messages on birthdays and HCT anniversaries, and social reunions of individuals who have undergone HCT.

### Power Calculation and Analysis Plan

For adequate power to capture a range of effect sizes in the final registry, we targeted a sample size that would be feasible to recruit and provide adequate statistical power to detect smaller effect sizes (eg, standardized mean difference [SMD]<0.3). To determine whether HCT survivors differ from matched nontransplanted patients with respect to pain, physical functioning, and HRQoL, we would need approximately 1000 patients (500 per group) to have at least 85% power to detect a 0.20 SMD in these outcome measures among the 2 groups using a 2-sided 2-sample *t* test with a type I error rate of 0.025. The primary analysis strategy relies on the use of a propensity score. It is possible that some patients may be missing important baseline data, restricting their inclusion in the propensity score analysis. However, even with 20% missing data without any imputation, our sample size would still achieve at least 80% power to detect a minimum effect size of 0.22 with a 0.025 type I error rate.

Power was calculated using a 2-sample *t* test using Power Analysis & Sample Size (PASS) version 14.0.8 (NCSS, LLC, Kaysville, UT, USA). To recruit an adequate sibling cohort for post-HCT patients with SCD, we will enroll 500 HCT survivors, with nontransplanted patients with SCD recruited at a 1:1 ratio. Assuming at least 75% of patients with HCT will have a sibling control, 375 post-HCT patients with SCD with 375 sibling controls would provide at least 80% and 85% power to detect minimum effect sizes of 0.23 and 0.24, respectively, in the sibling cohort samples using a 2-sided 2-sample *t* test with a 0.025 significance level.

An estimated 996 cases of HCT for SCD were reported between 2008 and 2017, and annually over 140 new cases are reported to the CIBMTR [5,43]. The centers participating in this study reported nearly half of all HCT procedures reported to the CIBMTR to date. Further, since reporting of HCT to the CIBMTR was not mandatory before 2008, and some of the participating centers do not currently report data to the CIBMTR, there may be additional patients available for study. Thus, for this study of the feasibility of the registry, we estimated that if we can enroll and capture outcomes on 50% of the eligible individuals, we would have demonstrated the feasibility of adequate enrollment of post-HCT participants.

## Results

### Participant Details

We attempted to contact 100 eligible post-HCT individuals with SCD, identified from a review of medical records at a single center. We enrolled 72 (72%) post-HCT individuals who were 9-38 (median 17) years old and 1-29 (median 3) years old, and 63 (87%) of them had received the transplant from an HLA-identical sibling donor. After optimizing study procedures for transplant recipients, we sequentially opened the study to enroll siblings without SCD and nontransplanted patients with SCD. To date, we have enrolled 19 siblings aged 5-32 (median 10) years and have also enrolled 28 nontransplanted controls with SCD, aged 4-46 (median 22) years. Of the 119 participants enrolled in the study so far, 85 sets of surveys have been completed by 73 (61%) nonduplicated participants (51 [70%] post-HCT, 11 [15%] siblings, 11 [15%] controls), including 80 completed PROMIS HRQOL surveys. Although there was variability in the numbers who started each of the different surveys, there were few missing data fields overall and they appeared random for the completed surveys. A total of 44 (61%) post-HCT subjects with SCD (26 [59%] females, 18 [41%] men), 10 (53%) sibling donors, and 10 (36%) nontransplanted controls with SCD have completed the fertility-screening surveys. In addition, hormone surveys have been completed by 63 (53%) individuals, offspring surveys by 22 (19%) individuals, and surveys on PROMIS satisfaction with sexual function by 21 (18%) participants. Measurements of height and weight were available on 72 (61%) individuals. To date, 41 (34%) participants have provided research blood samples, and 20 (17%) participants have also submitted samples on subsequent time points. We piloted data retrieval from the CIBMTR for post-HCT subjects with SCD enrolled in Atlanta, GA, USA. We requested data on 52 post-HCT participants in this study on whom data had previously been submitted to the CIBMTR; 11 (21%) subjects had not signed consent for research at the original data submission to the CIBMTR, so no data could be shared. Of 41 participants who had provided consent to the CIBMTR for using their data for research, 14 (34%) had limited essential transplant data collected, while 27 (66%) had been randomized to gather detailed research case report forms. The missing data from the CIBMTR were completed by abstracting data from electronic medical records. The study is now opening at centers in the U.S., U.K., Nigeria, and Canada, and enrollment at other centers has commenced. We have completed 28 (24%) American Time Use Survey (ATUS) interviews.

## Discussion

### Principal Findings

We described the design and implementation of an international registry designed to capture long-term and late effects of HCT for SCD. Although the importance of long-term and late effects of HCT for SCD is well recognized and consensus guidelines for follow-up have been published, there is still a lack of data on the subject [44-47].

Initial implementation of STELLAR at the pilot site in Atlanta, GA, USA, suggests that such a study is feasible on a large scale. We recruited 72 (72%) of 100 eligible post-HCT patients with SCD at a single site even as we iteratively implemented optimization of our methodology. Enrollment continues, although it is impacted by interruptions in routine clinic attendance during the COVID-19 pandemic. Nevertheless, this invitation response rate for recruitment is comparable to the recruitment experience of long-term survivors of HCT in the BMTSS [48], although the participants are predominantly African Americans and may be disadvantaged by health disparities. Further, although several consenting patients did not complete surveys, those who started surveys completed them with minimal missing data.

### Comparison With Previous Studies

This study was modeled on the extensively validated methodology of the Childhood Cancer Survivor Study and the BMTSS [24,49,50]. These studies have refined, validated, and implemented approaches to studying of late effects of treatment. Therefore, we adopted the best practices in the field for this study and adapted them for electronic data capture by patients using a computer, tablet, or smartphone. In addition, we added validated measures of PROs, physical function, and vital measurements.

### Strengths

The CIBMTR captures data on the survival of transplant recipients through the HCT center lifelong. The CIBMTR has also demonstrated that centralized PRO data collection in HCT is feasible and clinically meaningful [51]. However, individuals with stable donor-derived erythropoiesis post-HCT are typically no longer followed at the HCT center or to the center. This is likely because they are no longer perceived as needing any specialized services, are not eligible for Medicaid because they do not have the diagnosis of SCD (for US participants), live far from the center, or have difficulty obtaining health insurance as adults. Thus, the HCT center may not be able to provide data or subject access to the CIBMTR. In addition, their primary care provider may or may not be aware of current guidelines on monitoring for the late effects of HCT and may not be performing the screening procedures.

Further, since PROs are not captured during routine clinical encounters, they cannot be retrieved by mining electronic medical records. STELLAR is thus designed to supplement the efforts of the CIBMTR by directly engaging post-HCT and control study participants and enrolling and prospectively following participants in the long term. Such an approach can provide granular information about the health status, health behaviors, and health outcomes throughout the life course.

Another major strength of this study is the detailed follow-up of sexual function and fertility potential. Ovarian and testicular dysfunction are significant concerns for patients facing gonadotoxic therapies. The strong likelihood of loss of reproductive potential is a substantial consideration of patients and their caregivers considering HCT [52]. In addition, sexual dysfunction is a concern in patients post-HCT [53], especially females with failure to produce sex hormones. Males with SCD who have had recurrent priapism are at risk for sexual dysfunction. Therefore, it is crucial to understand the prevalence of infertility, low sex hormone production, and sexual dysfunction in all patients with SCD, including after HCT and autologous GT. These understudied outcomes will aid in counseling patients about expectations around reproductive health with or without curative therapy. In addition, assessing patient perceptions of risk for infertility or sexual dysfunction will assist in adapting communication about reproductive health to avoid inaccurate patient perceptions of reproductive health risks secondary to HCT. An accurate understanding of patient perceptions will help decision-making and promote decisional satisfaction.

### Key Lessons Learned in Implementation

In implementing STELLAR at the coordinating site in Atlanta, GA, USA, we learned several vital lessons about recruiting and retaining participants that helped us refine our approach. First, after trying various strategies to reach out to post-HCT patients, we found that the ex-sickle clinic, a clinic focused on the long-term follow-up of survivors of HCT, provides the best opportunity to approach potential participants. The majority of the HCT and sibling participants were enrolled and study procedures were carried out in the setting of an ex-sickle clinic. Such a clinic allows study data to be collected contemporaneously to deliver clinical care and, thus, minimizes participant burden. Further, a sizable majority of post-HCT patients have undergone the procedure within the past 8 years [5]. Therefore, they are likely to retain connections to the HCT center. Thus, they could potentially be reached at such a clinic.

Second, in piloting the study, it became apparent that completing several surveys and repeating them annually may pose a substantial participant burden. Therefore, we have successfully implemented several measures to reduce participant burden, including using branching logic, splitting surveys into multiple parts, and allowing survey data to be carried over from year to year, with the participant being able to review and accept or edit the responses. Several patients who consented to participate in the study still did not complete PROs, which underscores the difficulties inherent in understanding what motivates participants to consent and remain in a registry in the long term, how to optimize their experience, and how to communicate information derived from the study that is of interest to them.

### Limitations

There are several limitations to this study. First, we recruited participants who remained connected to a single HCT center with a well-established long-term follow-up in an ex-sickle clinic. Expanding this study to the other US and international centers with different institutional and cultural settings with various health care models may require overcoming barriers that may not have been foreseen. Even at this single center, we were only able to offer the study to those patients for whom we had a current address and who were responsive to our efforts to reach them. Thus, our sample does not include any patients unwilling to or unable to connect to the HCT center. Second, we have available PROs only from participants who completed them. Thus, we do not know the impact of selection bias and missing data on STELLAR. To minimize this potential bias and reach a more significant proportion of transplant recipients, we have refined our approach to recruiting subjects directly through internet and social media advertisements. An emerging body of literature will guide our efforts to implement and refine web-based recruitment and address the ethical, regulatory, and logistical issues related to the recruitment and retention of study participants online [54-57]. Once we enroll subjects online, we will also seek their consent to contact their HCT center and obtain additional, detailed, and accurate clinical information relevant to the study.

### Conclusion

We described the design of STELLAR and the feasibility of capturing outcomes in patients with SCD who have undergone HCT or GT, their siblings without SCD, and nontransplanted patients with SCD. We also reported the critical lessons learned from refining the study design and optimizing study processes at the lead site in Atlanta, GA, USA. In addition, we have taken essential steps to establish methods for retrieving data submitted to the CIBMTR and harmonizing data collection. Thus, STELLAR provides a model for the longitudinal collection of critical data on the long-term outcomes of HCT and contemporaneous comparison cohorts, which are vital for future studies of allogeneic HCT and autologous GT. The necessary next steps will be the participation of sites worldwide, with ongoing feasibility evaluation of multisite participation.

## Appendix

Multimedia Appendix 1 Peer review summary statement from National Insttutes of Health (NIH).

## Abbreviations

AMH: anti-Mullerian hormone
ASCQ-Me: Adult Sickle Cell Quality of Life Measurement Information System
ATUS: American Time Use Survey
BMTSS: Bone Marrow Transplant Survivor Study
cGVHD: chronic graft-versus-host disease
CIBMTR: Center for International Blood and Marrow Transplant Research
DFCI: Dana Farber Cancer Institute.
EMA: ecological momentary assessment
FSH: follicle-stimulating hormone
GT: gene therapy
GVHD: graft-versus-host disease
HCT: hematopoietic cell transplantation
HLA: human leukocyte antigen
HRQOL: health-related quality of life
IRB: Institutional Review Board
LH: luteinizing hormone
PROMIS: Patient-Reported Outcomes Measurement Information System
SCD: sickle cell disease
SCD SEQ-C: Sickle Cell Disease Self-Efficacy Questionnaire for Children
SMD: standardized mean difference
STELLAR: Sickle Cell Transplantation Evaluation of Long-Term and Late Effects Registry
TUG: timed up and go
