# Supplementary material for: Sickle Cell Transplantation Evaluation of Long-term and Late Effects Registry (STELLAR) to Compare Long-term Outcomes After Hematopoietic Cell Transplantation to Those in Siblings Without Sickle Cell Disease and in Nontransplanted Individuals With Sickle Cell Disease: Design and Feasibility Study
Source: JMIR Res Protoc. 2022 Jul 6;11(7):e36780. doi: 10.2196/36780 (PMC9301564; doi:10.2196/36780)
Supplement: Multimedia Appendix 1 [file resprot_v11i7e36780_app1.pdf]

SUMMARY STATEMENT
(Privileged Communication)

PROGRAM CONTACT:
Nahed El Kassar
301-495-0054
nahed.elkassar@nih.gov

Release Date: **03/18/2019**
Revised Date:

Application Number: **1 R01 HL141882-01A1**

Principal Investigator

KRISHNAMURTI, LAKSHMANAN

Applicant Organization: EMORY UNIVERSITY

Review Group: **CHSA**
Cancer, Heart, and Sleep Epidemiology A Study Section

Meeting Date: **02/21/2019**
Council: **MAY 2019**
Requested Start: **07/01/2019**
RFA/PA: **PA18-484**
PCC: **BB N**

Project Title: **Sickle Cell Post Transplantation Long Term and Late Effects Registry (STELLAR)**

SRG Action: **Impact Score:33 Percentile:23**
Next Steps: **Visit https://grants.nih.gov/grants/next_steps.htm**
Human Subjects: 30-Human subjects involved - Certified, no SRG concerns
Animal Subjects: 10-No live vertebrate animals involved for competing appl.
Gender: 1A-Both genders, scientifically acceptable
Minority: 2A-Only minorities, scientifically acceptable
Children: 1A-Both Children and Adults, scientifically acceptable

Project Year	Direct Costs Requested	Estimated Total Cost
1	1,514,999	2,129,842
2	1,515,000	2,129,843
3	1,515,000	2,129,843
4	1,515,000	2,129,843
5	1,515,000	2,129,843
TOTAL	7,574,999	10,649,215

ADMINISTRATIVE BUDGET NOTE: The budget shown is the requested budget and has not been adjusted to reflect any recommendations made by reviewers. If an award is planned, the costs will be calculated by Institute grants management staff based on the recommendations outlined below in the COMMITTEE BUDGET RECOMMENDATIONS section.

1R01HL141882-01A1 KRISHNAMURTI, LAKSHMANAN

RESUME AND SUMMARY OF DISCUSSION: The investigators plan to evaluate the late effects and long-term outcomes of hematopoietic cell transplantation (HCT) for sickle cell disease (SCD). They will build on established national collaborations to assemble a cohort of 500 survivors of transplantation for SCD and compare them with 500 age-, sex, and sickle cell type matched untransplanted SCD patients, and 500 sibling bone marrow donors. The committee agreed the scientific foundation for the proposed study was supported by strong preliminary data. They thought it was significant because it has potential to address gaps in knowledge regarding the health consequences associated with HCT for SCD. The reviewers discussed additional strengths including the strong team of investigators, and the creation of the biorepository. Although the reviewers thought the investigators had been responsive to the previous critiques, there were remaining weaknesses in the rigor of the approach for each aim. There were concerns with the feasibility of recruiting and following the controls, inadequate discussion on strategies for retention of individuals who participate, inadequate justification for the number of centers, and lack of clarity regarding the data collection. Overall, despite the weaknesses, the panel considered this a strong application and expects it could have a high impact on the field.

DESCRIPTION (provided by applicant): Hematopoietic cell transplantation (HCT) remains the only curative treatment for sickle cell disease (SCD), an inherited red blood cell disorder characterized by substantial morbidity, poor health related quality of life (HRQoL) and premature mortality. HLA-identical sibling donor HCT results in 94% overall and 91% event-free survival. Clinical trials and registries limit data collection to HCT related outcomes and do not capture long-term clinical, or patient reported data or bio-specimens necessary to study late effects or include comparison to controls. Late complications of HCT include gonadal dysfunction, secondary neoplasms, impairment of certain HRQoL domains, immune dysregulation, and cardiovascular disease (CVD), based mostly on observations of patients who underwent HCT for malignant diseases. SCD patients undergoing HCT for SCD are not exposed to some of these risk factors for late effects since they have mostly received matched sibling donor HCT in childhood. They may however have multi-organ organ damage, and impaired HRQoL before HCT which may influence long-term outcomes. There is a paucity of data about long term outcomes of HCT for SCD. The increasing applicability of HCT through the use of alternative donors, and gene therapy provide an urgent imperative for an evidence base for the long-term comparative effectiveness of HCT. Our long-term goal is to improve outcomes of HCT for SCD, our objective is to determine the long-term outcomes of HCT for SCD. We have established collaborations with the Center for International Bone Marrow Transplant Research (CIBMTR) In North American consortium, we will assemble a cohort of 500 survivors of HCT for SCD, an equal number of un-transplanted SCD patients, matched for age, gender, and phenotype and sibling bone marrow donors, in a prospective registry to address critical knowledge gaps. We have created, pilot-tested, and refined the infrastructure, and generated preliminary data to support the feasibility of recruitment and performing study procedures. Our overarching hypothesis is that HCT for SCD leads to improved HRQoL and immune function but is associated with gonadal damage, impaired sexual function. We propose the following specific aims to test the hypothesis: 1. To ascertain the impact of HCT on HRQoL, pain, costs, utilization, finances and employment. 3. To determine the effect of HCT on gonadal and sexual function in HCT survivors. 4. To ascertain splenic regeneration, immune reconstitution, response to pneumococcal vaccines and T-cell repertoire post HCT. These aims will generate a critical evidence base for the impact of HCT on long-term outcomes of SCD, inform decision making, and guide future research to prevent or reverse late effects of HCT in SCD and other non-malignant diseases.

PUBLIC HEALTH RELEVANCE: This study will assemble a North American cohort of survivors of transplantation for sickle cell disease, for the first time, to study the late effects of transplantation and to compare the long-term outcomes following transplantation with that in un-transplanted patients as well as healthy controls. The results of this study are likely to generate the evidence base to guide decision making by patients and clinicians and inform future research on prevention of late effects and improving outcomes of transplantation for sickle cell disease.

CRITIQUE 1

Significance: 2
Investigator(s): 1
Innovation: 2
Approach: 4
Environment: 2

Overall Impact: This amended application by an outstanding team of investigators proposes to evaluate the late effects and long-term outcomes of hematopoietic cell transplantation (HCT) for sickle cell disease (SCD). The investigators will build on established national collaborations to assemble a cohort of 500 survivors of transplantation for SCD and compare them with 500 age-, sex, and sickle cell type matched untransplanted SCD patients, and 500 sibling bone marrow donors. The study will collect information on health-related quality of life (HRQoL), pain, financial distress, gonadal function, fertility potential, sexual function, and splenic function. The scientific premise is strong, and the study addresses clinically important gaps. SCD is the most common inherited blood disorder in the US (occurs in ~1 in 500 African Americans) and results in substantial morbidity and premature mortality. Currently, HCT is the only curative treatment for SCD. However, the long-term outcomes of HCT are largely unknown. Clinical trials and registries collect outcomes related to HCT including short-term event-free survival but information on long-term outcomes including patient reported outcomes are lacking. The PI is a pediatric hematology oncologist with over 23 years of research experience in SCD. He is PI of a U01 comparing standard of care to HCT for SCD patients. The other investigators bring expertise in cancer survivorship, patient reported health outcomes, biobanking, biostatistics and recruitment into longitudinal studies. Addressing concerns from previous reviews they now include expertise in urology, reproductive endocrinology, and fertility preservation. The study will also establish a biorepository, which is another strength. Pilot work has been conducted to demonstrate the ability to identify HCT survivors and controls. They will use a variety of methods to track HCT survivors. Many of the previous concerns have been addressed. However, feasibility to recruit and enroll at other centers is still not demonstrated. Additionally, lack of clarity in the approach around the aims and data collection still remains.

1. Significance:

Strengths

- SCD is the most common inherited blood disorder in the US (occurs in ~1 in 500 African Americans) and results in substantial morbidity, poor health related quality of life (HRQoL), and premature mortality.
- Currently, HCT is the only curative treatment for SCD.
- There are limited data on long-term outcomes of HCT for SCD including limited information on HRQoL as well as gonadal function and fertility potential.
- These gaps limit the applicability and acceptability of HCT for SCD treatment. The findings from this study would help to address these knowledge gaps.

Weaknesses

- Appear to be very small samples sizes for Aim 2 (gonadal and sexual function) and Aim 3 (splenic regeneration, immune reconstitution, response to pneumococcal vaccines)

2. Investigators:

Strengths

- Strong team experienced in HCT for SCD.
- Dr. Krishnamurti (PI) is a pediatric hematologist oncologist with over 23 years of research experience in SCD. He is the Director of the Pediatrics BMT program. Currently, he is PI of an NHLBI U01 grant for a multicenter study of comparing standard of care to HCT for SCD.
- The other Co-Is bring expertise in cancer survivorship, patient reported health outcomes, pediatric endocrinology, fertility preservation, pathogenesis of sickle cell disease, biobanking, graft rejection, biostatistics, recruitment into longitudinal studies.

Weaknesses

- None noted

3. Innovation:

Strengths

- Comparing outcomes in transplanted patients with their sibling HLA identical bone marrow donors (potentially share same genetic and environmental influences).
- Most studies of HCT for SCD have not systematically collected HRQoL and pain data and only at one-year post HCT; the proposed study will capture data over the long-term as well as at 1 year post-HCT which will allow us to understand the impact of complete recovery following HCT as well as the impact of late sequelae on patient reported outcomes PROs.
- Establishing a biorepository.

Weaknesses

- None noted.

4. Approach:

Strengths

- Using a variety of methods to track HCT survivors and limiting to large HCT centers initially (can expand if needed).
- Preliminary data indicated that contact was established with 94% of HCT survivors and access to siblings was also achieved.
- Using a variety of methods to collect PROs and biospecimens (online web-based surveys that can be completed via computer, tablet or phone; paper option available as well; home based specimen collection at convenient times)
- Created methods and mechanisms to engage investigators, participants and the general public (presentations, annual reunions for survivors and their families, use of Facebook) to facilitate recruitment.
- Ability to identify and match a high number of untranslated SCD controls.
- Employing several analytic approaches (matching, propensity scoring, multivariable regression) to attempt to overcome selection bias and balance known confounders.
- Established an external advisory committee including experts in HCT, SCD and patients.

Weaknesses

- Although the ability to identify and match controls using existing data was shown, the ability to recruit and follow the controls was not demonstrated.
- Although a large number of transplant centers (30 to begin with) will be included, it’s not clear how many of the 1217 post-HCT patients are still alive. What are the annual survival rates?

- Home based phlebotomy is mentioned but nothing is mentioned about the physical function measures (hand grip, walk test) being conducted in the home and how that would be done systematically and who would perform these measures (trained study staff or mobile phlebotomy techs?).
- It is not clear what is meant by EMA pain data and whether this is completed only by children or everyone recruited for Aim 1.
- Email reminders are sent for surveys, but it is not clear if reminders are sent for completing the pain diary.
- Aims seem to be including different individuals. Age ranges are not specified for each aim. It is not clear who will be asked to complete the PROs, provide the biospecimens to compare gonadal function and fertility potential, questionnaires that measure perceptions of the risk for infertility.
- Aim 2 sample size/power section states 30 males, 30 female HCT; 30 males/30 females on HU, and 10 males/10 females on chronic PRBC transfusion.
- Aim 3 mentions that 250 patients with a diverse range of age will be included as well as a convenience sample of 30 HCT patients, 30 donors and 30 SCD controls.

5. Environment:
Strengths

- Well established infrastructure at CHOA.

Weaknesses

- None noted.

Protections for Human Subjects:

Acceptable Risks and/or Adequate Protections

Data and Safety Monitoring Plan (Applicable for Clinical Trials Only):

Not Applicable (No Clinical Trials)

- Included but is relevant to a clinical trial rather than an observational study.

Inclusion of Women, Minorities and Children:

- Sex/Gender: Distribution justified scientifically
- Race/Ethnicity: Distribution justified scientifically
- For NIH-Defined Phase III trials, Plans for valid design and analysis:
- Inclusion/Exclusion of Children under 18: Including ages <18; justified scientifically
- Children ages 3 and older will likely be enrolled; this is justified given the epidemiology of SCD

Vertebrate Animals:

Not Applicable (No Vertebrate Animals)

Biohazards:

Not Applicable (No Biohazards)

Resubmission:

- Largely responsive but a lack of clarity in the approach around the aims and data collection still remains.

Applications from Foreign Organizations:

Not Applicable (No Foreign Organizations)

Select Agents:

Not Applicable (No Select Agents)

Resource Sharing Plans:

Unacceptable

- Not included

Budget and Period of Support:

Recommend as Requested

CRITIQUE 2

Significance: 2
Investigator(s): 2
Innovation: 3
Approach: 4
Environment: 2

Overall Impact: This proposal is a resubmission of a prior R01. This study assembles and investigates a large cohort of 1+ year survivors of BMT for Sickle Cell Disease. It proposes to assemble a large retrospective group using information from the IBMTR and 37 primary institutions; it will also create a much smaller prospective cohort. The investigations proposed on QOL, health, fertility and immune function would have tangible impact on transplant decisions and follow up strategies for patients with SCD. These possibilities are tempered by the structure of the grant; in particular the large number of centers and interest in expanding those further, lack of information on the feasibility of the project outside of Children's Hospital of Atlanta, and the unsure future of the cohort. While the resubmission does address some of the concerns of the prior review many remain.

1. Significance:

Strengths

- BMT is the only curative therapy for SCD
- Utilization of BMT has increased as has survival following BMT
- There is limited QOL and overall health data following BMT for SCD
- Understanding the late outcomes may clarify decision making for future patients

Weaknesses

- Cohort is constructed but not clear how successful a prospective cohort it will be

2. Investigators:

Strengths

- Strong PI in Dr. Krishnamurti
- Strong group of collaborators with diverse skills including cohort studies, endocrinology, immunology
- Group is focused at Emory

Weaknesses

- Non-identified

3. Innovation:

Strengths

- Use of multiple sources for patient identification
- Innovative strategies for selection of control groups

Weaknesses

- None noted

4. Approach:

Strengths

- Collaboration with the IBMTR
- Use of multiple sites to broaden the cohort size

Weaknesses

- The complexity of the study would argue for a more limited number of centers. The criteria of 10 transplants (ever?) is given, but it is not clear how much gain there is at the low end of accrual vs select higher volume centers
- No rationale is provided for the expansion to even more centers
- The assumption that tracking and participation rates experienced at CHOA would be more convincing if substantiated by data from other major participants
- There is little information provided on strategies for retention of individuals who participate
- The prospective nature of the study is not well defined

5. Environment:

Strengths

- Strong organization structure is available at Emory/CHOA

Weaknesses

- Distributed nature of the grant makes it likely that there will be weak collaborators

Protections for Human Subjects:

Acceptable Risks and/or Adequate Protections

Data and Safety Monitoring Plan (Applicable for Clinical Trials Only):

Not Applicable (No Clinical Trials)

Inclusion of Women, Minorities and Children:

- Sex/Gender: Distribution justified scientifically
- Race/Ethnicity: Distribution justified scientifically
- For NIH-Defined Phase III trials, Plans for valid design and analysis: Not applicable
- Inclusion/Exclusion of Children under 18: Including ages <18; justified scientifically

Vertebrate Animals:
Not Applicable (No Vertebrate Animals)

Biohazards:
Not Applicable (No Biohazards)

Resubmission:

- The applicants have addressed many of the concerns raised in the initial grant but the proposal is going to be very logistically complicated and it is not clear how they will be able to retain the cohort

Applications from Foreign Organizations:
Not Applicable (No Foreign Organizations)

Select Agents:
Not Applicable (No Select Agents)

Resource Sharing Plans:
Not Applicable (No Relevant Resources)

Authentication of Key Biological and/or Chemical Resources:
Not Applicable (No Relevant Resources)

Budget and Period of Support:
Recommend as Requested

CRITIQUE 3

Significance: 2
Investigator(s): 2
Innovation: 2
Approach: 5
Environment: 2

Overall Impact: This is a resubmission of a grant examining the impact of hematopoietic cell transplantation on patients with sickle cell disease by comparing transplanted patients with sibling donors, matched controls, and baseline values over time. The grant examines outcomes ranging from health-related quality of life, pain scales, financial stress (AIM 1), to gonadal function and fertility potential (AIM 2), and immune reconstitution, splenic regeneration, response to pneumococcal. The applicants leverage a novel network of sites that have transplanted these patients and novel collaborations. The applicants have been responded to the prior application by increasing information on feasibility of enrollment and have bolstered preliminary findings. Feasibility is also aided by simplification of numbers of sites involved. The prior grant had significantly dampened enthusiasms because of the rigor in the approach section which is greatly improved, but still has multiple flaws within each aim, mostly related to the focused on multiple outcomes. The different challenges in the analyses of each are not well addressed (prelim data shows changes in some outcomes but not others, but the rationale for why these should change in the full study is not clear). Financial strain is actually hypothesized to change over time, but this is not specified in the analysis or power calculation (additionally the issue of assessing this outcome across siblings and potential correlation of financial stressors not addressed). Overall, these are multiple minor weakness because within each aim several outcomes are strongly justified and supported by prior data. The importance of the research question and the unique study design and access of patients is a major strength.

THE FOLLOWING SECTIONS WERE PREPARED BY THE SCIENTIFIC REVIEW OFFICER TO SUMMARIZE THE OUTCOME OF DISCUSSIONS OF THE REVIEW COMMITTEE, OR REVIEWERS' WRITTEN CRITIQUES, ON THE FOLLOWING ISSUES:

PROTECTION OF HUMAN SUBJECTS: ACCEPTABLE

INCLUSION OF WOMEN PLAN: ACCEPTABLE

INCLUSION OF MINORITIES PLAN: ACCEPTABLE

INCLUSION OF CHILDREN PLAN: ACCEPTABLE

COMMITTEE BUDGET RECOMMENDATIONS: The budget was recommended as requested.

Footnotes for 1 R01 HL141882-01A1; PI Name: KRISHNAMURTI, LAKSHMANAN

NIH has modified its policy regarding the receipt of resubmissions (amended applications). See Guide Notice NOT-OD-14-074 at http://grants.nih.gov/grants/guide/notice-files/NOT-OD-14-074.html. The impact/priority score is calculated after discussion of an application by averaging the overall scores (1-9) given by all voting reviewers on the committee and multiplying by 10. The criterion scores are submitted prior to the meeting by the individual reviewers assigned to an application, and are not discussed specifically at the review meeting or calculated into the overall impact score. Some applications also receive a percentile ranking. For details on the review process, see http://grants.nih.gov/grants/peer_review_process.htm#scoring.

MEETING ROSTER

Cancer, Heart, and Sleep Epidemiology A Study Section
Population Sciences and Epidemiology Integrated Review Group
CENTER FOR SCIENTIFIC REVIEW
CHSA
02/21/2019 - 02/22/2019

Notice of NIH Policy to All Applicants: Meeting rosters are provided for information purposes only. Applicant investigators and institutional officials must not communicate directly with study section members about an application before or after the review. Failure to observe this policy will create a serious breach of integrity in the peer review process, and may lead to actions outlined in NOT-OD-14-073 at https://grants.nih.gov/grants/guide/notice-files/NOT-OD-14-073.html and NOT-OD-15-106 at https://grants.nih.gov/grants/guide/notice-files/NOT-OD-15-106.html, including removal of the application from immediate review.

CHAIRPERSON(S)

NORTH, KARI E, PHD
PROFESSOR
DEPARTMENT OF EPIDEMIOLOGY
SCHOOL OF PUBLIC HEALTH
UNIVERSITY OF NORTH CAROLINA AT CHAPEL HILL
CHAPEL HILL, NC 27514

MEMBERS

AMANKWAH, ERNEST, PHD, MS *
ASSISTANT PROFESSOR
DEPARTMENT OF ONCOLOGY
SIDNEY KIMMEL COMPREHENSIVE CANCER CENTER
JOHNS HOPKINS UNIVERSITY SCHOOL OF MEDICINE
TAMPA, FL 33647

ARMSTRONG, GREGORY, MD
MEMBER
DEPARTMENT OF EPIDEMIOLOGY & CANCER CONTROL
ST. JUDE CHILDREN'S RESEARCH HOSPITAL
MEMPHIS, TN 38105

BIBBINS-DOMINGO, KIRSTEN, MD, PHD
PROFESSOR & LEE GOLDMAN, MD ENDOWED CHAIR
DEPARTMENT OF EPIDEMIOLOGY AND BIOSTATISTICS
UNIVERSITY OF CALIFORNIA, SAN FRANCISCO
SAN FRANCISCO, CA 94143

BROECKEL, ULRICH, MD *
PROFESSOR OF PEDIATRICS
CHIEF OF GENOMIC PEDIATRICS
DEPARTMENT OF PEDIATRICS
MEDICAL COLLEGE OF WISCONSIN
MILWAUKEE, WI 53226

CARLSON, CHRISTOPHER S, PHD
ASSOCIATE MEMBER
CANCER PREVENTION PROGRAM
FRED HUTCHINSON CANCER RESEARCH CENTER
SEATTLE, WA 98109

CHEN, YU, PHD
ASSOCIATE PROFESSOR
DIVISION OF EPIDEMIOLOGY
DEPARTMENT OF POPULATION HEALTH
NEW YORK UNIVERSITY SCHOOL OF MEDICINE
NEW YORK, NY 10016

FIGUEIREDO, JANE C, PHD *
ASSOCIATE PROFESSOR
DEPARTMENT OF MEDICINE
SAMUEL OSCHIN COMPREHENSIVE CANCER INSTITUTE
CEDARS-SINAI MEDICAL CENTER
LOS ANGELES, CA 90048

GIDDING, SAMUEL S, MD
MEDICAL DIRECTOR
FH FOUNDATION
PASEDENA, CA 91106

GUALLAR, ELISEO, MD, DRPH
PROFESSOR
DEPARTMENT OF EPIDEMIOLOGY
BLOOMBERG SCHOOL OF PUBLIC HEALTH
JOHNS HOPKINS UNIVERSITY
BALTIMORE, MD 21205

HAIMAN, CHRISTOPHER ALAN, SCD
PROFESSOR
DEPARTMENT OF PREVENTIVE MEDICINE
KECK SCHOOL OF MEDICINE
UNIVERSITY OF SOUTHERN CALIFORNIA
LOS ANGELES, CA 90089

HENDERSON, TARA OLIVE, MD *
ASSOCIATE PROFESSOR
DEPARTMENT OF PEDIATRICS
THE UNIVERSITY OF CHICAGO MEDICINE
CHICAGO, IL 60637

HERNANDEZ, BRENDA Y, PHD *
ASSOCIATE PROFESSOR AND DIRECTOR
HAWAII TUMOR REGISTRY
CANCER RESEARCH CENTER OF HAWAII
UNIVERSITY OF HAWAII
HONOLULU, HI 96813

HIXSON, JAMES E, PHD *
PROFESSOR
HUMAN GENETICS CENTER
DEPARTMENT OF EPIDEMIOLOGY
SCHOOL OF PUBLIC HEALTH
UNIVERSITY OF TEXAS HEALTH SCIENCE CENTER
HOUSTON, TX 77030

LANGE, ETHAN MATHER, PHD
PROFESSOR
DIVISION OF BIOMEDICAL INFORMATICS AND
PERSONALIZED MEDICINE
DEPARTMENT OF MEDICINE
UNIVERSITY OF COLORADO - ANSCHUTZ MEDICAL CAMPUS
AURORA, CO 80045

LEVY, PHILLIP DAVID, MD, MPH
PROFESSOR AND ASSOCIATE CHAIR
DEPARTMENT OF EMERGENCY MEDICINE
ASSISTANT VP FOR TRANSLATIONAL SCIENCE AND
CLINICAL RESEARCH INNOVATION
WAYNE STATE UNIVERSITY
DETROIT, MI 48201

LI, YI, PHD
PROFESSOR
DIRECTOR OF CHINA INITIATIVES
DEPARTMENT OF BIOSTATISTICS
UNIVERSITY OF MICHIGAN
ANN ARBOR, MI 48109

LOPEZ, DAVID S, DRPH *
ASSISTANT PROFESSOR
DEPARTMENT OF PREVENTIVE MEDICINE
UTMB SCHOOL OF MEDICINE
GALVESTON, TX 77555

MA, XIAOMEI, PHD
PROFESSOR
DEPARTMENT OF CHRONIC DISEASE EPIDEMIOLOGY
SCHOOL OF PUBLIC HEALTH
YALE UNIVERSITY
NEW HAVEN, CT 06520

MICHAUD, DOMINIQUE S, SCD
PROFESSOR
DEPARTMENT OF PUBLIC HEALTH &
COMMUNITY MEDICINE
TUFTS UNIVERSITY MEDICAL SCHOOL
BOSTON, MA 02111

NAN, BIN, PHD *
PROFESSOR
DEPARTMENT OF STATISTICS
UNIVERSITY OF CALIFORNIA, IRVINE
IRVINE, CA 92697

NEGLIA, JOSEPH P, MD, MPH *
PROFESSOR AND HEAD
DEPARTMENT OF PEDIATRICS
UNIVERSITY OF MINNESOTA
MINNEAPOLIS, MN 55455

OCHS-BALCOM, HEATHER M, PHD *
ASSOCIATE PROFESSOR
DEPARTMENT OF EPIDEMIOLOGY AND
ENVIRONMENTAL HEALTH
SCHOOL OF PUBLIC HEALTH AND HEALTH PROFESSIONS
UNIVERSITY BUFFALO AT BUFFALO
BUFFALO, NY 14214

PAJUKANTA, PAIVI, MD, PHD
PROFESSOR
DEPARTMENT OF HUMAN GENETICS
DAVID GEFFEN SCHOOL OF MEDICINE
UNIVERSITY OF CALIFORNIA, LOS ANGELES
LOS ANGELES, CA 90095

PATIL, SUJATA, PHD *
ASSOCIATE ATTENDING BIOSTATISTICIAN
DEPARTMENT OF EPIDEMIOLOGY AND BIOSTATISTICS
MEMORIAL SLOAN KETTERING CANCER CENTER
NEW YORK CITY, NY 10065

REYNOLDS, KRISTI, PHD *
RESEARCH SCIENTIST AND DIRECTOR
EPIDEMIOLOGIC RESEARCH
DEPARTMENT OF RESEARCH AND EVALUATION
KAISER PERMANENTE
PASADENA, CA 91101

ROHATGI, ANAND KUMAR, MD *
ASSOCIATE PROFESSOR
DEPARTMENT OF INTERNAL MEDICINE/ CARDIOLOGY
UT SOUTHWESTERN MEDICAL CENTER
DALLAS , TX 75390

SHAH, SANJIV J, MD
STONE ENDOWED PROFESSOR
DIVISION OF CARDIOLOGY
DEPARTMENT OF MEDICINE
FEINBERG SCHOOL OF MEDICINE
NORTHWESTERN UNIVERSITY
CHICAGO, IL 60611

SHERMAN, MARK E, MD *
PROFESSOR
MAYO CLINIC
JACKSONVILLE, FL 32224

SONG, YIQING, SCD, MD
PROFESSOR
RICHARD M FAIRBANKS SCHOOL OF PUBLIC HEALTH
INDIANA UNIVERSITY
INDIANAPOLIS, IN 46202

STEINMAUS, CRAIG M, MD, MPH
PUBLIC HEALTH MEDICAL OFFICER
OFFICE OF ENVIRONMENTAL HEALTH HAZARD
ASSESSMENT
CALIFORNIA ENVIRONMENTAL PROTECTION AGENCY
OAKLAND, CA 94720

SUNDERRAM, JAG, MBBS
PROFESSOR
DIVISION OF PULMONARY AND CRITICAL CARE MEDICINE
DEPARMENT OF MEDICINE
ROBERT WOOD JOHNSON MEDICAL SCHOOL
RUTGERS UNIVERSITY
NEW BRUNSWICK, NJ 08903

TAMIMI, RULLA M, SCD
ASSOCIATE PROFESSOR
CHANNING DIVISION OF NETWORK MEDICINE
BRIGHAM & WOMEN'S HOSPITAL
BOSTON, MA 02115

WANG, WENYI, PHD
ASSOCIATE PROFESSOR
DEPARTMENT OF BIOINFORMATICS AND
COMPUTATIONAL BIOLOGY
MD ANDERSON CANCER CENTER
HOUSTON, TX 77030

WILKINSON, JAMES D, MD *
PROFESSOR
SCHOOL OF MEDICINE
VANDERBILT UNIVERSITY
NASHVILLE, TN 37232

SCIENTIFIC REVIEW OFFICER

WIESCH, DENISE, PHD
SCIENTIFIC REVIEW OFFICER
CENTER FOR SCIENTIFIC REVIEW
NATIONAL INSTITUTES OF HEALTH
BETHESDA, MD 20892

EXTRAMURAL SUPPORT ASSISTANT

ESCOBEDO ARANGO, MAURICIO
LEAD EXTRAMURAL SUPPORT ASSISTANT
CENTER FOR SCIENTIFIC REVIEW (CSR)
POPULATION SCIENCES AND EPIDEMIOLOGY (PSE) IRG
NATIONAL INSTITUES OF HEATH (NIH)
BETHESDA, MD 20892

* Temporary Member. For grant applications, temporary members may participate in the entire meeting or may review only selected applications as needed.

Consultants are required to absent themselves from the room during the review of any application if their presence would constitute or appear to constitute a conflict of interest.
